# Alteration of the aggregation and spatial organization of the vector of Chagas disease, *Triatoma infestans*, by the parasite *Trypanosoma cruzi*

**DOI:** 10.1038/s41598-019-53966-w

**Published:** 2019-11-22

**Authors:** Stéphanie Depickère, Gonzalo Marcelo Ramírez-Ávila, Jean-Louis Deneubourg

**Affiliations:** 1 Laboratorio Entomología Médica, INLASA, La Paz, Bolivia; 2 Instituto de Investigaciones Físicas, Universidad Mayor de San Andrés, La Paz, Bolivia; 3 CENOLI, Université Libre de Bruxelles, 1050 Brussels, Belgium; 4 Present Address: The Abdus Salam, International Centre for Theoretical Physics (ICTP), Trieste, Italy

**Keywords:** Behavioural ecology, Dynamical systems, Animal behaviour, Entomology

## Abstract

Triatominae insects are vectors of the parasite *Trypanosoma cruzi*, the etiological agent of Chagas disease affecting millions of people in Latin America. Some species, such as *Triatoma infestans*, live in the human neighborhood, aggregating in walls or roof cracks during the day and going out to feed blood at night. The comprehension of how sex and *T. cruzi* infection affect their aggregation and geotaxis is essential for understanding their spatial organization and the parasite dispersion. Experiments in laboratory-controlled conditions were carried out with groups of ten adults of *T. infestans* able to explore and aggregate on a vertical surface. The influence of the sex (male vs. female) and the proportion of infected insects in the group were tested (100% of infected insects vs. a small proportion of infected insects, named infected and potentially weakly infected groups, respectively). Therefore, four distinct groups of insects were tested: infected males, infected females, potentially weakly infected males, and potentially weakly infected females, with 12, 9, 15, and 16 replicates, respectively. The insects presented a high negative geotaxis and a strong aggregation behavior whatever the sex or their infection. After an exploration phase, these behaviors were stable in time. The insects exhibited a preferential vertical position, head toward the top of the setup. Males had a higher negative geotaxis and a higher aggregation level than females. Both behaviors were enhanced in groups of 100% infected insects, the difference between sexes being maintained. According to a comparison between experimental and theoretical results, geotaxis favors the aggregation that mainly results from the inter-attraction between individuals.

## Introduction

Chagas disease is one of the most important neglected tropical diseases with 6–7 million worldwide people who are estimated to be infected, and 20% of the population of endemic areas who are at risk^1–3^. This vector-borne disease is caused by the parasite *Trypanosoma cruzi* (Kinetoplastida: Trypanosomatidae) and is mainly transmitted by contact with infected feces/urine of hematophagous insects of the Triatominae subfamily (Hemiptera: Reduviidae). Currently, more than 150 species have been described worldwide, and all of them are considered as potential vectors^4–9^. Most of the species live in sylvatic habitats, and only a dozen of species are regarded as vectors of major epidemiological importance due to their capacity to live in the surrounding of the human dwellings where they find stable shelters and food abundance^10^. *Triatoma infestans* is the main vector in the Southern Cone of South America; they are known to be easily infected by *T. cruzi* generating its common use in the detection of parasites in Chagasic patients and domestic animals infection through xenodiagnosis^11,12^. Except for their feeding specialization, the domiciliary species share similar lifestyle and cycle of activities with many gregarious arthropods including other synanthropic species like cockroaches^13–15^. During the daytime, they assemble in dark and sheltered places such as cracks in the walls or roofs, or behind objects hanging on walls. At night, they leave their shelter to actively seek a host upon which to feed and then, they come back to a resting place to digest. The digestive phase can last from some days to several weeks according to the blood meal size, the individual, the nymphal stage and the environmental conditions^16^.

The control strategy for Chagas disease relies mainly on the control of the domestic vectors through chemical control^1^. Faced with the increased insecticide resistance exhibited by these insects, and with the reinvasion of the dwellings by residual or sylvatic populations of triatomines^17–19^, it is necessary to study the behaviors leading to a better understanding of the distribution and the dispersion of vectors. In this perspective, aggregation and geotaxis are key behaviors. Knowing these behaviors better and understanding how the parasites may influence the insects is fundamental. Indeed, aggregation is a widespread behavior that results from a response of individuals to environmental heterogeneity, and from interactions involving attractions between individuals^20–22^. The interactions between individuals maintain the group cohesion and the associated adaptive values of group living. In triatomines, protection against predation is usually evoked as the main benefice of clustering, but surviving might also be enhanced thanks to protection against hydric loss, and to a higher probability of coprophagy, symbiont exchange, and of sex encounters, as it was shown for other insects^23–27^. Aggregation in triatomines was investigated with a focus towards the substances that mediate it, and on the factors that modulate the aggregative response^28–33^. All these works analyzed nymphal instars behavior response; in adults, very few is known except that they can aggregate around feces^32^ and around mating pairs^34^. Geotaxis, also called gravitaxis, is a crucial behavior involved in insect orientation^35^. Animals can exhibit locomotion that is gravitationally directed vertically down or up (positive or negative geotaxis, respectively). Geotaxis in triatomine has been poorly described, *T. infestans* was just reported as being more concentrated in the upper half of the walls in houses or chicken houses^24,36^. Moreover, to our knowledge, no studies were conducted to analyze the synergy or conflict between gregariousness and geotaxis in triatomines.

It is well-known that parasites can modify physiological, behavioral, and/or morphological traits of their hosts to increase their fitness, even if it is at the cost of the host fitness^37^. The latter usually means that infected hosts will behave in ways that facilitate the transmission of the parasite^38,39^. Literature about the effects and possible manipulation of triatomines behavior by *T. cruzi* is relatively sparse, covering only seven species: *Mepraia spinolai, Panstrongylus megistus, Rhodnius pallescens, R. prolixus, T. brasiliensis, T. dimidiata* and *T. infestans*. Authors have been especially interested in the parasite’s effects on four groups of the host’s behavior: life-history trait, feeding, defecation, and dispersion/locomotion. It seems that *T. cruzi* increases the development time and biting rate, and decreases the longevity and defecation time in *M. spinolai*^40,41^. It also increases the development time and decreases the longevity in *R. prolixus*, a temperature-dependent effect^42^, but no effect was observed in the feeding and defecation behavior^43^. Finally, no change was observed in *P. megistus*^44^, *T. dimidiata*^45^, *T. infestans*^46^, and almost no change in *T. brasiliensis*^47^. The reproduction was decreased by *T. cruzi* in *T. brasiliensis*^47^; it was increased or decreased in *R. prolixus* according to the insect age and the rearing temperature^48^. The dispersion was higher in infected females of *T. dimidiata* than in non-infected females; no effect was found in males^49^. Moreover, *T. dimidiata* individuals infected with *T. cruzi* were found to have larger wings than non-infected ones^50^. In *R. pallescens*, *T. cruzi* infection did not significantly impact flight initiation, but infected females flew significantly faster than males from 30 s to 2 min after flight initiation^51^. The locomotory activity of *R. prolixus* was decreased by infection: the total number of movements was 20% less than that observed in non-infected insects^52^. The time to find a host for an infected *M. spinolai* was almost twice as fast as for a non-infected insect^41^. In conclusion, modification of the triatomine traits seems to be species-dependent, age-dependent, sex-dependent, and even environment/physiology-dependent.

In this work, video-recorded experiments were conducted where ten adults of *T. infestans* were dropped at the base of a vertical wall covered with a paper sheet allowing the bugs to climb. Spatial positions of each insect were extracted from the video every five minutes until 150 minutes, permitting the following of the dynamics and the calculation of the size and spatial stability of the clusters. Four groups of experiments were analyzed according to the sex (males vs. females) of insects and their *T. cruzi-*infection determined by microscopy (potentially weakly infected insects vs. infected insects, as it is explained in detail in Methods).

## Results

### Negative geotaxis: spatial distribution and total population

**Figure 1:**
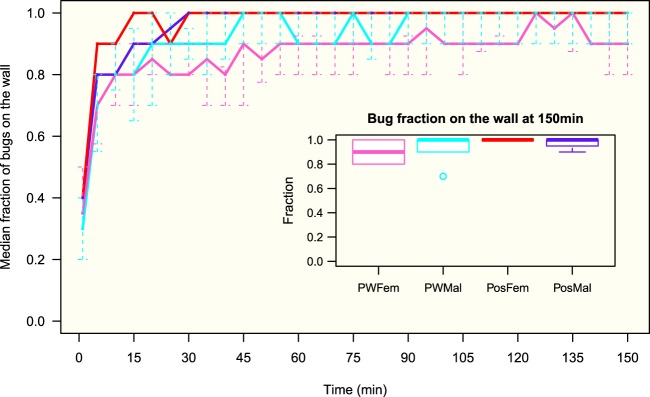
Median fraction of bugs on the wall (quantiles 25–75%). Number of experiments: 16, 15, 9, 12 for potentially weakly infected females (PWFem, pink), potentially weakly infected males (PWMal, cyan), infected females (PosFem, red) and infected males (PosMal, blue) respectively. Inserted figure: boxplot distribution of bugs on the wall at 150 min. Anderson-Darling test at 150 min between the 4 groups: *TkN* = −0.12, *P* = 0.46.

**Figure 2:**
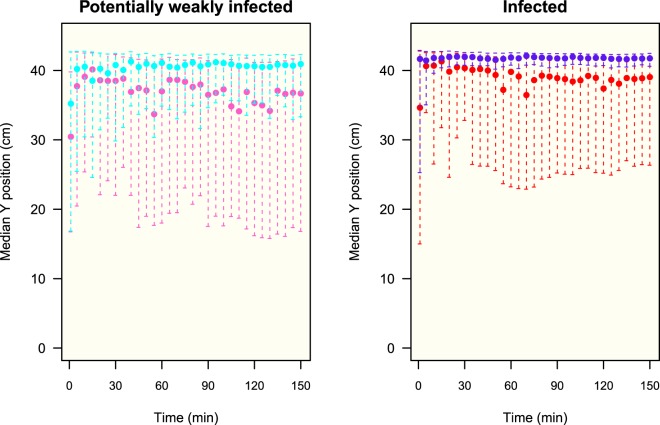
Evolution of the median vertical position (quantiles 25–75%) of the potentially weakly infected and infected insects on the wall during the experiment. Median position at 150 min: potentially weakly infected females (pink): 36.7 (16.8–41.2) cm, infected females (red): 39.0 (26.3–42.0) cm, potentially weakly infected males (cyan): 40.9 (33.3–42.3) cm, infected males (blue): 41.8 (40.6–42.5) cm.

**Figure 3:**
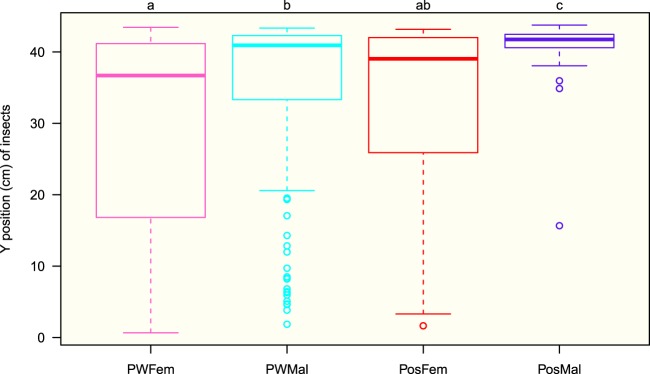
Boxplot distribution of the vertical position of the individuals at 150 min. Number of observations: potentially weakly infected females (PWFem): 145; potentially weakly infected males (PWMal): 141; infected females (PosFem): 90; infected males (PosMal): 117. Anderson-Darling k-sample test between the four groups for all individuals: *TkN* = 24.4, *P* < 0.001. Results of Anderson-Darling all-pairs comparison tests are shown at the top of the figure (groups with different letters correspond to groups statistically different at *P* < 0.001).

The bugs quickly climbed on the wall and stayed there, demonstrating a high negative geotaxis; and after an exploratory phase, the insects began to cluster and rest (see Supplementary Fig. S1). After 10 min, in all four groups more than 80% of the individuals were on the wall (90% after 20 min); and this proportion remained constant until the end of the experiment where no statistical difference was detected between the four groups (Fig. 1). The bugs were mostly located in the upper half of the setup, 22–44 cm from the bottom; the median vertical position reached a plateau value (stationary state) after 15 min with a value greater than 35 cm for the four groups (Fig. 2). Their vertical distributions at 150 min (end of the experiment) revealed a statistically significant difference between sexes, e.g., males were located higher than females, and also between potentially weakly infected and infected individuals, e.g., infected males were higher than potentially weakly infected males (Fig. 3). These trends were also discovered considering the top strip of 4 cm (40–44 cm), a zone corresponding to 10% of the total area of the setup and where 56% (80%) of the potentially weakly infected (infected) males and 33% (44%) of the potentially weakly infected (infected) females were located (see Supplementary Fig. S2).

**Figure 4:**
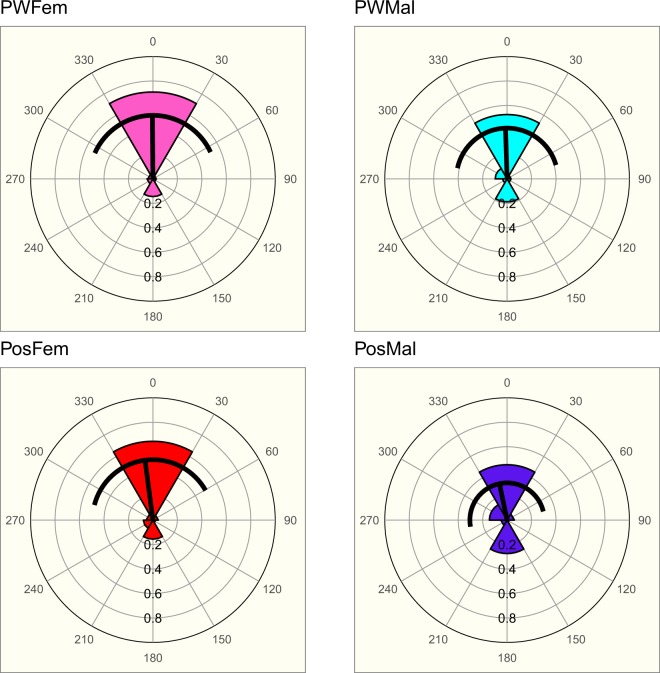
Vertical orientation of insects for the four groups. Wedge’s angles of 60°, frequencies are shown as radius of the wedge. 0° represents the head to the top. The dark line shows the mean direction and its length, and the standard deviation of the distribution. Number of observations: potentially weakly infected females (PWFem): 145, potentially weakly infected males (PWMal): 141, infected females (PosFem): 90 and infected males (PosMal): 117. Rao’s tests gave *P* < 0.01 for the four groups. V-tests (testing the null hypothesis of uniformity against non-uniform distribution with a mean of 0) gave *P* < 0.001 for the four groups. Mardia-Watson-Wheeler pairwise tests between the four groups: PWFem/PWMal: *P* = 0.24, PWFem/PosFem: *P* = 0.156, PWFem/PosMal: *P* = 0.001, PWMal/PosFem: *P* = 0.033, PWMal/PosMal: *P* = 0.005, PosFem/PosMal: *P* = 0.049.

At the end of the experiment, more than 80% of the individuals had a vertical orientation from which around 70–80% had the head turned towards the top of the setup (± 30°). For the four groups, individuals were not uniformly distributed (Rao’s test with *P* < 0.01), but rather centered on 0 (V-tests with *P* < 0.001, Fig. 4). When the distributions of individual orientations were compared, no difference appeared between sexes. Interestingly, the infection affected the orientation of the males which demonstrated a higher proportion of insects with the head towards the bottom when infected (Fig. 4).

To summarize, in potentially weakly infected groups, both sexes exhibited a high negative geotaxis that was higher for males than for females (Fig. 3). Most of the insects of both sexes were oriented the head toward the top (Fig. 4). The bug’s geotaxis was strengthened in groups of 100% infected insects, especially for males.

### Clustering

**Figure 5:**
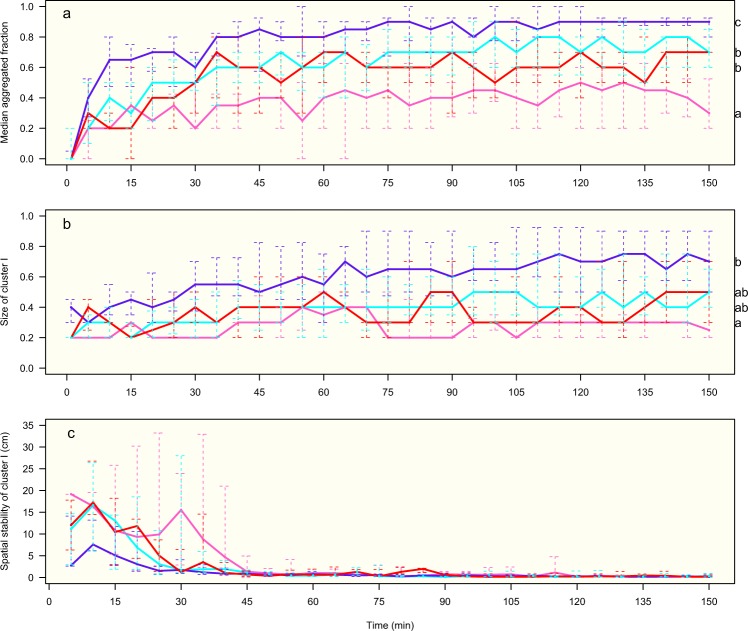
Dynamics of aggregation for the four groups: potentially weakly infected females (pink), potentially weakly infected males (cyan), infected females (red) and infected males (blue). (**a**) median fraction of aggregated individuals (quantiles 25–75%); (**b**) median size of the biggest cluster (quantiles 25–75%); (**c**) spatial stability of the biggest cluster (quantiles 25–75%). Anderson-Darling k-sample test at 150 min between the four groups: (**a**) *TkN* = 7.4, *P* < 0.001, (**b**) *TkN* = 4.9, *P* = 0.001, and (**c**) *TkN* = −0.5, *P* = 0.63. Results of Anderson-Darling all-pairs comparison test are shown with different letters corresponding to groups statistically different at *P* < 0.05 (on the right side of the figure).

**Figure 6:**
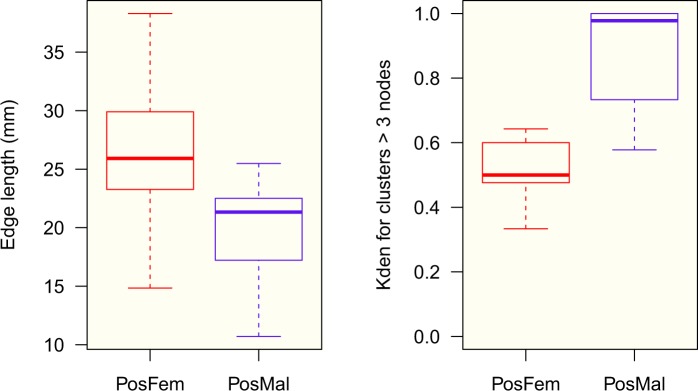
Structure of the clusters in infected groups. Boxplot of the size of edges (or links) between aggregated bugs (left) and K-density of the clusters of size >3 individuals (right). Anderson-Darling k-sample test for size of the edges: *TkN* = 9.1, *P* < 0.001 (72 and 255 observations for infected females (PosFem) and males (PosMal) respectively); and for the K-density: *TkN* = 4.7, *P* = 0.004 (17 and 21 observations for PosFem and PosMal respectively).

The median aggregated bug fraction increased up to reach a plateau around 35 min, gathering around 70% and 90% of potentially weakly infected and infected males respectively, and 40% and 60% of potentially weakly infected and infected females respectively (Fig. 5). A statistically significant difference was detected at 150 min between sexes (males showed a higher aggregated fraction than females), and between infected conditions (infected bugs with a higher aggregated level than potentially weakly infected ones) (Fig. 5, see also Supplementary Fig. S3). Insects in all groups tended to gather in one or two clusters. The biggest cluster assembled 40% (70%) of the aggregated potentially weakly infected (infected) population in males, and 30% (40%) of the aggregated potentially weakly infected (infected) population in females (Fig. 5). No difference was observed between sexes, neither between infection condition (Fig. 5). When the structure of the clusters was compared between 100% infected sexes, clusters of infected males looked more compact, with a significantly smaller distance between aggregated individuals; and they also looked denser, with a higher K-density (Fig. 6, see also Supplementary Fig. S4).

**Figure 7:**
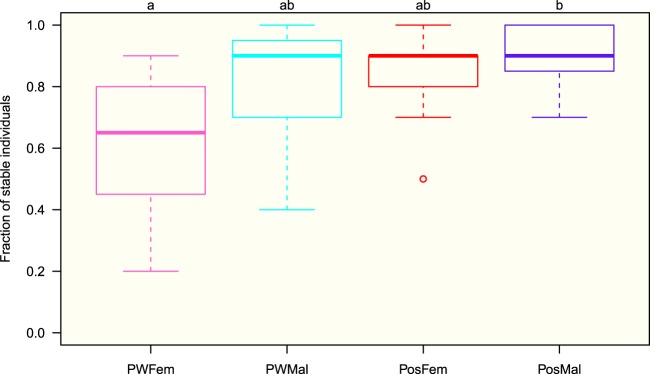
Boxplot of stable individuals (move <10 mm between two snapshots) for the four groups: potentially weakly infected females (PWFem) and males (PWMal), and infected females (PosFem) and males (PosMal). Anderson-Darling k-sample test between the four groups: *TkN* = 2.8, *P* = 0.017. Results of Anderson-Darling all-pairs comparison tests are shown at the top of the figure (different letters correspond to groups statistically significantly different at *P* < 0.005; same letters correspond to *P* > 0.05).

At the end of the experiments, individuals were very stable in space: the median fraction of individuals that moved less than 1 cm was greater than 60% for the four groups, and no difference between sexes and infection group was detected (Fig. 7). The biggest cluster also showed a strong spatial stability, with no statistically significant difference between the four groups (Fig. 5).

In order to verify that the fraction of aggregated individuals was not directly due to the method of calculating this fraction and the increase of the bugs density at the top of the setup, 20,000 repetitions of groups of *N* simulations were performed (*N* = 16 for PWFem, *N* = 15 for PWMal, *N* = 9 for PosFem, and *N* = 12 for PosMal). For each simulation, 10 points were vertically distributed following the experimental vertical distribution of the bugs at 150 min (see Supplementary Figure S5), and homogeneously horizontally distributed. For each group of simulations, the mean fraction of aggregated individuals was calculated for each repetition. The mean aggregated fractions obtained in the simulations were 0.26, 0.42, 0.36, and 0.72 for PWFem, PWMal, PosFem, and PosMal respectively, revealing that an increase of the geotaxis level leads to a rise in the observed aggregation level. However, the probability of observing a mean aggregated fraction higher or equal to the corresponding experimental one was *P* < 0.0001 for all the groups, demonstrating that the observed phenomenon involved an active aggregation due to the inter-attraction between individuals.

**Figure 8:**
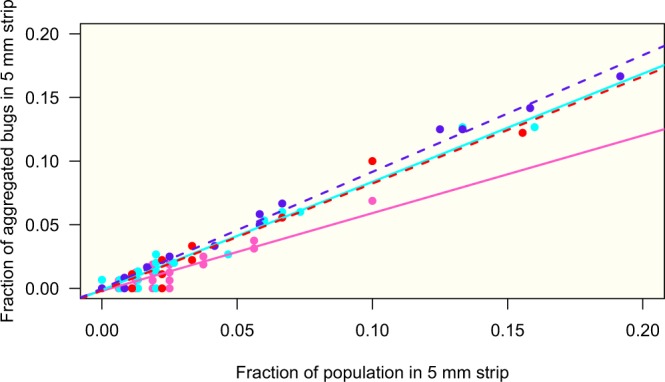
Fraction of aggregated individuals according to the population present in a horizontal strip of 5 mm high for the four groups: potentially weakly infected females (PWFem) and males (PWMal), and infected females (PosFem) and males (PosMal). Linear regressions: PWFem (pink): y = 0.6106x− 0.0019 (SE of slope: 0.0296), *R*² = 0.83, *P* < 0.001; PWMal (cyan): y = 0.8498x− 0.0013 (SE of slope: 0.0180), *R*² = 0.96, *P* < 0.001; PosFem (red): y = 0.8408x− 0.0017 (SE of slope: 0.0269), *R*² = 0.92, *P* < 0.001; PosMal (blue): y = 0.9168x− 0.0001 (SE of slope: 0.0076), *R*² = 0.99, *P* < 0.001.

The fraction of aggregated individuals in a strip of 0.5 cm was proportional to the fraction of the population settled in this strip (Fig. 8). The slope of the regression line was the lowest for the potentially weakly infected female group, and the highest for the infected male group, being intermediate and similar for the two other groups. The slopes of the linear regression were compared computing a model including the interaction between the total number of bugs and the groups: a significant interaction was found (*F*_3, 348_ = 30.04, *P* < 0.001), giving a slope equal to 0.61 for potentially weakly infected females, 0.85 for potentially weakly infected males, 0.84 for infected females, and 0.92 for infected males. The comparison of the slopes between the four groups showed that the potentially weakly infected females’ slope was lower than the three other groups’ slopes (*P* < 0.001), the infected males’ slope was higher than the three other groups’ slopes (*P* < 0.02), and the potentially weakly infected males’ slope was not different from the infected females’ slope (*P* = 0.98). These results demonstrated that, for the same density, the aggregation was higher for males than for females and for infected insects than for potentially weakly infected ones.

## Discussion

This work represents the first detailed analysis of aggregation and geotaxis in adult males and females of *T. infestans*, and how both sexes are affected by *T. cruzi* infection. As shown before in nymphal instars^53,54^, adults exhibited an active aggregation due to the inter-attraction between individuals. A stable aggregation emerged for both sexes, but the fraction of aggregated individuals and the density of the clusters were higher for males than for females. This difference between genders was maintained under *T. cruzi* infection, but the latter reinforced the gregariousness in both sexes.

Our results are in agreement with those of previous studies. Indeed, in a multi-factorial analysis (using species development stages and feces source altogether) of the aggregative response of individuals to feces the aggregation level was lower (but not statistically significantly different) for females than for males^32^. It is well established that clustering or reduction of the inter-individual distances of gregarious arthropods reduces various stresses as water loss, and energy consumption^25–27,55,56^. We hypothesize that the clustering of *T. infestans* individuals provides a similar benefit. Water loss is proportional to surface area that is proportional to the square of the size of the individual (length). The initial body water content is proportional to volume which is proportional to the cube of the individual size. Thus, relative water loss should be proportional to the surface area / volume ratio and decreases with the individual size implicating that bigger organisms have a higher resistance to dehydration^57–59^. As their weight and size are lower than females, males could be under higher hydric stress, leading them to a stronger aggregation. The same geometric hypothesis has been put forth to explain the reduction of water loss in an aggregate^26,27^. Moreover, it could be more adaptive for females to aggregate less to disperse their eggs and increase their probability of survival. In our experiments, males and females were supposed to be in similar physiological status due to their comparable period of starvation (8–10 days), but in the case of infection, *T. cruzi* and *T. infestans* compete for nutrients, and bug individuals show reduced resistance to starvation when they are infected^60^. It might be speculated that infected bugs were more starved and therefore exhibited a stronger aggregation to reduce the cost of the different stresses. Moreover, a higher negative geotaxis can indirectly contribute to improving the active aggregation by increasing the local densities of individuals.

We know very little about the distribution of triatomines inside a dwelling. In studies about the vertical distribution of *T. infestans* in Brazilian dwellings, adults, nymphs, and eggs of this species tended to be concentrated in the upper half of the wall^24^. In northern Argentina, 58.3% of a domestic *T. infestans* population obtained after the demolition of a house were in the roof, and eggs were aggregated at the top of the walls, decreasing gradually towards the floor (in^36^). In experiments using an artificial chicken house, *T. infestans* assembled at the upper part of the walls (in^36^). In the same way, in a setup simulating a chicken house, the post-feeding location of nymphs of *T. infestans* was mostly on the upper half of the walls even if four bunches of corn husks were placed on the ground as possible shelters^36^. Finally, in field studies in Bolivia, eggs and exuviae were easily found at the top of the walls, just at the beginning of the roof, especially in dwellings of the Andean valleys whose construction allows a wall-roof space (SD pers. obs.).

Detection of *T. cruzi* was realized by direct microscopic observation of drops of feces (see Methods). This is known as being less efficient than by polymerase chain reaction (PCR): microscopy can detect between 50% to 90% of what is identified as positive by PCR^61–68^. This variation can be caused by different factors such as the stage of the insect, the development of *T. cruzi* in the insect gut, the probes used in the PCR, etc. In our experiments, naturally infected adults captured in the field were used. They were maintained and fed in laboratory and examined for infections around 1.5–2 months after their arriving at the laboratory. Adults tend to be one of the most infected stages as the probability to get infection increases with the number of feedings, and so with age. Thus, it is more likely that the adults used in our study got infected time before their capture. It is consequently possible that a small proportion of microscopically negative insects – around 20% - would have been detected as infected by PCR. Based on this estimation, a simple binomial test shows that around 90% of our microscopically negative groups of 10 individuals contain three or less infected individuals. Despite this handicap, the difference observed between groups was statistically significant. In the same way, a part of the experiments with potentially weakly infected insects was composed of a mix of microscopically negative and positive insects (≤ 20%, see Methods). The latter implies that a small proportion of infected individuals inside a group of observed negative bugs is not enough to observe a change at the group level. Another interesting question is whether all the discrete typing units (DTUs) of *T. cruzi* and even strains of these DTUs, will influence the behavior of the bugs in a similar manner. Indeed, different strains of *T. cruzi* had different consequences in life history outcomes of *R. prolixus*^69^. A variation of the DTUs circulating in chagasic patients and triatomines can occur^70^. Nevertheless, the main DTUs circulating in a domestic/peridomestic context in the region of capture are from the group TcII/TcV/TcVI in chagasic patients^71,72^, as in domestic *T. infestans*^73,74^. Thus, more experiments are necessary to understand how *T. cruzi* affects the mechanisms underlying the geotaxis and the clustering, from a physiological and a behavioral point of view.

Behavioral alterations upon infection are called parasitic manipulation when they are adaptive for the parasite, altering phenotypic traits of its host in a way that enhances its probability of transmission. Some examples where the parasitism affects the geotaxis and the gregarious behavior of the hosts were described^75–80^. Is there any advantage to *T. cruzi* to enhance the negative geotaxis and the aggregation behavior of the triatomine? Several hypotheses can be proposed. On the one hand, a higher negative geotaxis can help to maintain the insect vector away from ground predators (mainly rodents^24,81^, and as suggested by experiments ducks and chicken^24,36,82^, and dogs^83^ in the case of domiciliated species like *T. infestans*), facilitate the triatomine mate-finding^81^ ensuring the reproduction of the insect vector as it was shown in cockroaches^84^, and amplify the formation of clusters by increasing the local densities of individuals. On the other hand, a stronger aggregation can improve the fitness of the triatomine through the reduction of the stress/energy consumption as it was demonstrated in various insects and discussed above. It can also allow a higher rate of coprophagy and cleptohaematophagy increasing the survival of the insect vector, especially the first nymphal instars, and also the probability of dispersion of parasites between triatomines^85^. Some works have reported a change in the locomotion/dispersion of the infected vectors: infected females of *T. dimidiata* have a higher dispersion on the field^49^, and infected nymphs of *R. prolixus* exhibited, on the contrary, a reduction of their locomotory activity especially at the beginning of the scotophase^52^. These changes in the infected vector locomotion/dispersion could affect the predation of the triatomes and the parasite dispersion. Indeed, in the wild, some animals like insectivorous mammals seem to have a relatively high probability of getting the infection through oral transmission by predation on triatomines^86–89^. Jansen *et al*. (2018) considered the transmission of *T. cruzi* by insect/flesh predation highly probable for a large number of mammal species^90^. However, more researches should be conducted to understand how the parasite influences the different behavior of the vectors, during both the scotophase and the photophase, to observe the effects on the transmission of the parasite.

A low height device like the setup used in these experiments allowed us to highlight differences, between sexes, and between groups with different proportion of infected insects. Knowledge about the spatial distribution of the insects in their natural conditions is scarce. The response of the bugs and their spatial distribution should be modulated according to many factors such as their development stages and their physiological condition. In adults, sexual attraction will also play a role in their distribution. In triatomines, the sex pheromone is emitted by the females, inducing males moving towards the females^16^. The air current present on a wall could allow the males to identify at some distance the females, as it was highlighted in domestic cockroaches, insects that have a similar way of life than triatomines except for the feeding habits^84^. In our experiments, shelters were deliberately missing to study the inter-attraction at the base of the gregariousness. Nevertheless, shelters are known to be the center of the clustering^16^. Similarly to the cockroaches and other gregarious insects, the individual response to the refuge could favor the mix of the insects whatever the category they belong to^91,92^. So, to help to control/monitor *T. infestans*^93^, it is important to design new experiments to study how the interplay between the behavior of this insect, infected or not, the spatial distribution of the shelters and of the blood sources, and the climatic conditions influence the spatial distribution of the triatomines.

## Methods

*T. infestans* specimens were collected in dwellings from Yacuiba Municipality (Gran Chaco region), Department of Tarija, Bolivia, in the area Tierras Nuevas (S21.748334, W63.561866, 621 m asl) - San Francisco de Inti (S21.818193, W63.588042, 600 m asl). They were maintained at the Medical Entomology laboratory of the National Institute of Laboratories in Health (INLASA) which directly depends on the Bolivian Ministry of Health, in La Paz, Bolivia. Bugs were kept in the insectarium maintained at 26 ± 1 °C, 60 ± 15% RH, 12: 12 night: dark cycle, using a system of electric heaters with thermostat and timer, humidifiers, and a bulb light controlled by a timer. The insects were kept at a density of ~30 adults in rectangular plastic jar of 2 liters with wide mouth containing a folded piece of kraft paper (40 cm × 15 cm) commonly used in the insectarium and closed with a piece of tulle held in place by a rubber band. They were fed on hens once every three weeks, following the relevant guidelines and regulations of the INLASA. About two months after the transfer to the laboratory, natural infection of insects by *T. cruzi* was determined by the analysis of drops of feces under a light microscope. This simple and low-cost method was also chosen for experimental reason: the insect must be kept alive. The infection rate of the captured insects was 47.5 ± 21.7%. As in the zone the risk of encountering insects infected with other species of *Trypanosoma* or trypanosomatid-like organisms is very low (0.1%^68^), we can consider it as negligible and suppose that all infected insects were infected by *T. cruzi*. Four conditions resulting from the combination between sex, using males and females, and two proportions of infected insects were then studied. In infected groups, all the males (abbreviated PosMal in Figures, 12 replicates) and females (PosFem, 9 replicates) were observed as infected by microscopy. Regarding the potentially weakly infected groups, fifteen and sixteen experiments were carried out with microscopically *T. cruzi-*negative males and females, respectively. Because the detection of *T. cruzi* by microscopy can generate false negative results, mainly for insects with a low-density parasitemia (microscopy detects between 50% to 90% of what is identified as positive by PCR^61–68^, see Discussion), we cannot exclude that these groups included some infected insects. Moreover, due to an accidental mixture by the technician in charge in the insectarium, nine of these experiments (four and five experiments in males and females respectively) could actually include some certainly infected insects. Therefore, a test for *T. cruzi* infection of the insects from these nine experiments was realized again by microscopy, determining a proportion of the infected individuals being less or equal to 20%. At 150 min, the aggregated fraction from these nine experiments was closer to the fraction found in potentially weakly infected groups than to the fraction observed in infected groups (Anderson-Darling k-sample test: *TkN* = 6.08, *P* < 0.001; number of experiments: infected: 12 (9) for infected males (females), 100% microscopically negative: 11 (11) with 100% microscopically negative males (females), 80% microscopically negative: 4 (5) with ≤20% infected males (females); Anderson-Darling all-pairs comparison test: 100% microscopically negative males vs. 80% microscopically negative males: *P* = 0.91; 80% microscopically negative males vs. infected males: *P* = 0.08; 100% microscopically negative females vs. 80% microscopically negative females: *P* = 0.45; 80% microscopically negative females vs. infected females: *P* = 0.03). Therefore, for each sex, the weakly infected groups and the 100% microscopically negative groups were aggregated, and named potentially weakly infected males and females, respectively (PWMal, PWFem). Experiments were conducted from around two months after the arrival of the insects at the laboratory, the time necessary to be acclimatized to the laboratory, physiologically homogenized, and to be tested for their infection by *T. cruzi*.

### Setup and methods

A glass aquarium was used (50 × 20 × 50 cm) to avoid escaping of *T. infestans* which is unable to climb on glass walls. Insects were allowed to climb on one of the vertical surfaces of this aquarium (50 × 50 cm) offered by a paper sheet (kraft paper, 43 × 44 cm). The glass setup was washed, and the paper changed at the end of each experiment. It was illuminated by a centered 60 W incandescent light bulb, placed at 50 cm behind the wall covered by the paper sheet. The paper guaranteed a homogeneous illumination of the setup; insects receiving around 60 lx. A video camera (Sony DCR-SR68) placed in front of the setup recorded the bug activity for 150 min. A 1 m high polystyrene wall surrounded the setup to isolate it. Experiments were conducted in a quiet and dark room to avoid any disturbance, at the beginning of the photophase. The temperature, relative humidity, and time from the beginning of the photophase for each experiment are given in Supplementary Material S6. No significant difference in experimental conditions was detected between the groups at a threshold of 0.05 (Supplementary Material S6). Ten bugs (8–10 days of starvation) were dropped on the bottom of the setup. They explored their environment rapidly and climbed on the wall. From the recordings, a snapshot was extracted at 1 min, 5 min and then every 5 min up to 150 min (31 snapshots in total). A processing program allowed us to record the spatial position of the thorax of each bug on each snapshot. With these spatial coordinates, the inter-individual distances were computed. As the length of an adult is on average 2.5 cm, and due to a tactile (legs or antennae) or visual perception, two individuals were considered as aggregated when they were at a distance less or equal to 4 cm.

The different groups were tested randomly between males and females, assuring the non-dependence of the results to the day of their testing. Furthermore, linear regressions were done to observe if the number of aggregated insects from one side, and the mean vertical position of the insect at the end of the experiment from another side depended on the time of the testing, and no trend was found (*P* > 0.05). As the experiments covered both climatic seasons of the Chaco (dry season - from end of April to end of November, and humid season), a Chi-squared test was done showing that no difference appeared in the number of experiments in each season between the four groups (*χ*² = 0.49, df = 3, *P* = 0.92). Finally, no correlation was found between the aggregation and geotaxis results and the conditions of temperature, relative humidity, and time from the beginning of the photophase (Supplementary Material S6).

### Indices and statistics

Several indices of position and aggregation were calculated using processing programs: 1) the number of individuals on the paper sheet; 2) the number of aggregated individuals; 3) the number and the size of the clusters; 4) the spatial stability of the individual (% of individuals that were found at time *t* + 1 in a circle of 10 mm in radius centered on the coordinate of the insect at time *t*); 5) the spatial stability of the biggest cluster (study of the distance between the centroid of the biggest cluster at time *t* + 1 and the centroid of the biggest cluster at time *t*). Finally, the individual position of the insects in the setup at the end of each experiment was analyzed, recording the vertical orientation of the bugs (position 0: head towards the top), to put forward a privileged position. A vertical orientation was defined as inside an angle of ± 30° to the vertical, head oriented towards the top or the bottom. Outside this range, the insect was not considered in a vertical position anymore. The structure of the clusters was also compared between infected males and infected females, groups where bigger clusters emerged. Each cluster was considered as an undirected network where each node represented an individual. Links between nodes were established when the distance between them was less or equal to 4 cm (the threshold for considering aggregation). The cluster K-density (ratio of the number of edges divided by the number of possible edges) was compared for clusters with a size greater than three individuals.

The comparisons between groups were made using the Anderson-Darling k-sample test^94^. In case of obtaining a *P* < 0.05, an Anderson-Darling all-pairs comparison test was performed. These statistics were calculated using the functions adKSampleTest and adAllPairsTest of the *PMCMRplus* package of R^95,96^. Circular statistics were carried out with Oriana 4.02 (Kovach Computing services). Uniformity of data was tested using Rao’s test, mean comparisons using V-test, and distribution comparisons using Mardia-Watson-Wheeler pairwise test. The structure of the clusters was analyzed using the *igraph* package in R^97^. The linear regression was done using the lm function, and the comparison of the slopes of regression with the *lsmeans* package of R, using Least-squared means (predicted marginal means)^98^. The boxplot used as a representation of the data distribution gives the median as the midline, the first and third quartiles as lower and upper limits of the box, respectively. The whiskers extend up to 1.5 times the interquartile range from the top (bottom) of the box to the furthest datum within that distance. Any data beyond that distance are represented individually as points.

## Supplementary information

Supplementary material

## Data Availability

The datasets generated during and/or analyzed during the current study are available from the corresponding author on reasonable request.
