# Supplementary material for: Alteration of the aggregation and spatial organization of the vector of Chagas disease, Triatoma infestans, by the parasite Trypanosoma cruzi
Source: Sci Rep. 2019 Nov 22;9:17432. doi: 10.1038/s41598-019-53966-w (PMC6874570; doi:10.1038/s41598-019-53966-w)

Alteration of the aggregation and spatial organization of the vector of Chagas disease, *Triatoma infestans*, by the parasite *Trypanosoma cruzi*

Stéphanie Depickère, Gonzalo Marcelo Ramírez-Ávila, Jean-Louis Deneubourg

Figure S1. Examples of insect's positions at 150 min in the setup for the four conditions

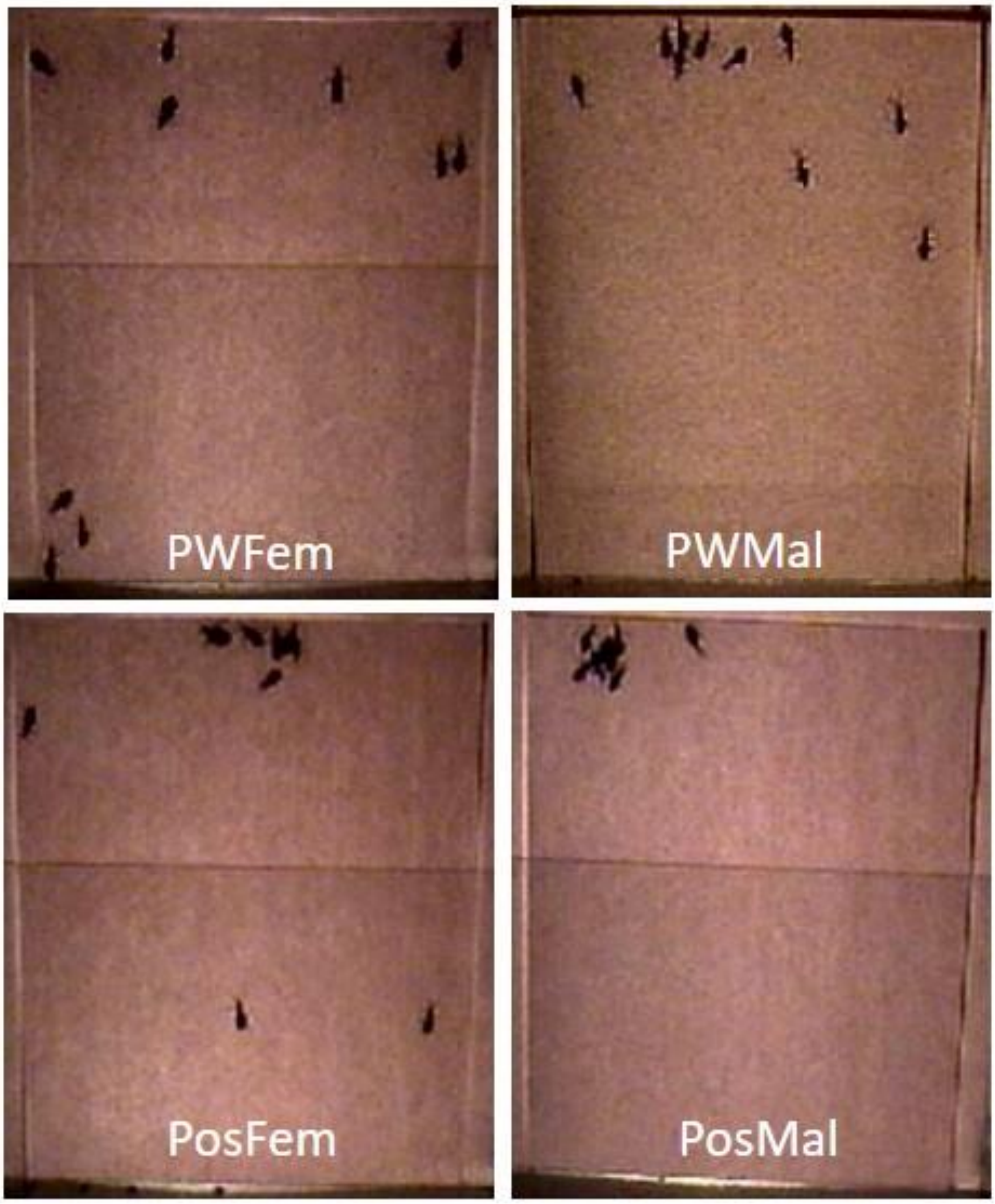

Figure S2. Boxplot distribution of the median fraction of individuals at the top of the setup (40-44 cm) at 150 min. Number of observations: 16, 15, 9, 12 for PWFem, PWMal, PosFem, and PosMal respectively. Anderson-Darling k-sample test between the four conditions for all individuals: $TkN = 8.0$, $P < 0.001$. Results of Anderson-Darling all-pairs comparison tests are shown at the top of the figure (conditions with different letters correspond to conditions statistically different at $P < 0.04$)

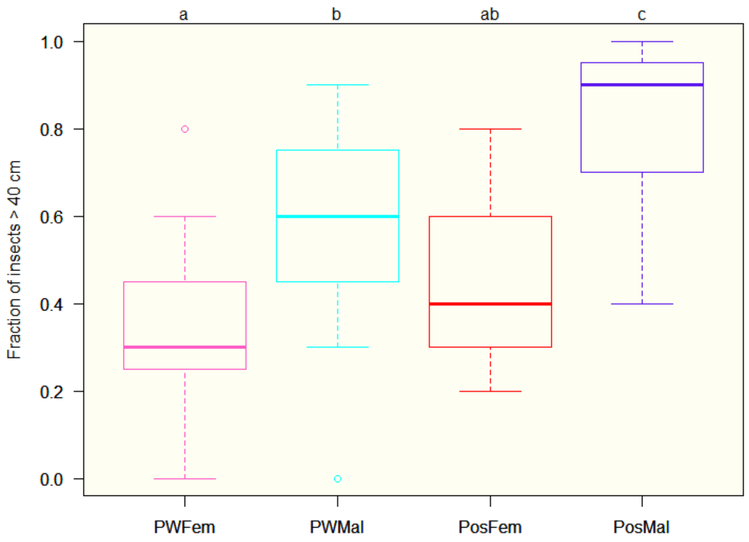

Figure S3. Relative frequency of experiments according to the aggregated fraction of individuals for the four conditions

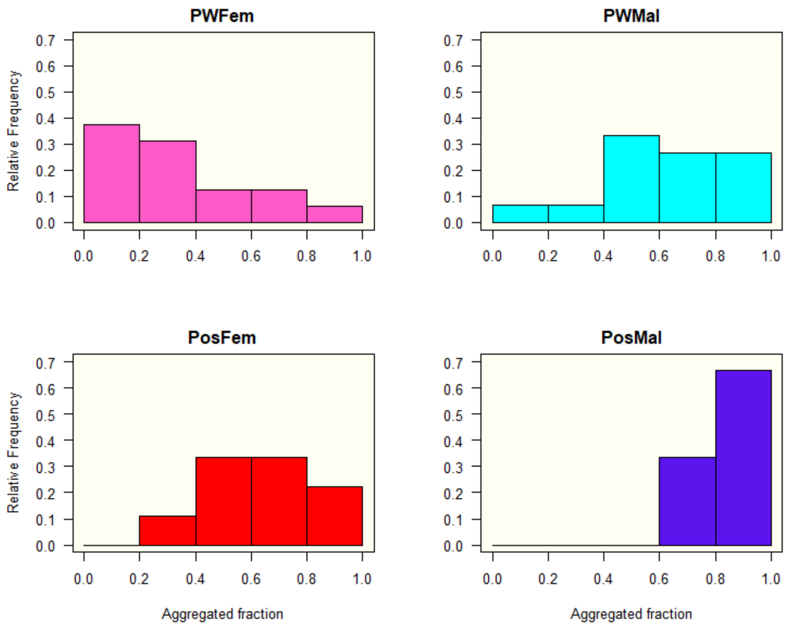

Figure S4. Example of clusters of 10 bugs in infected conditions. Nodes represent the insect positions on the wall. Links between nodes are present only when the distance is less or equal to 4 cm (the aggregative distance)

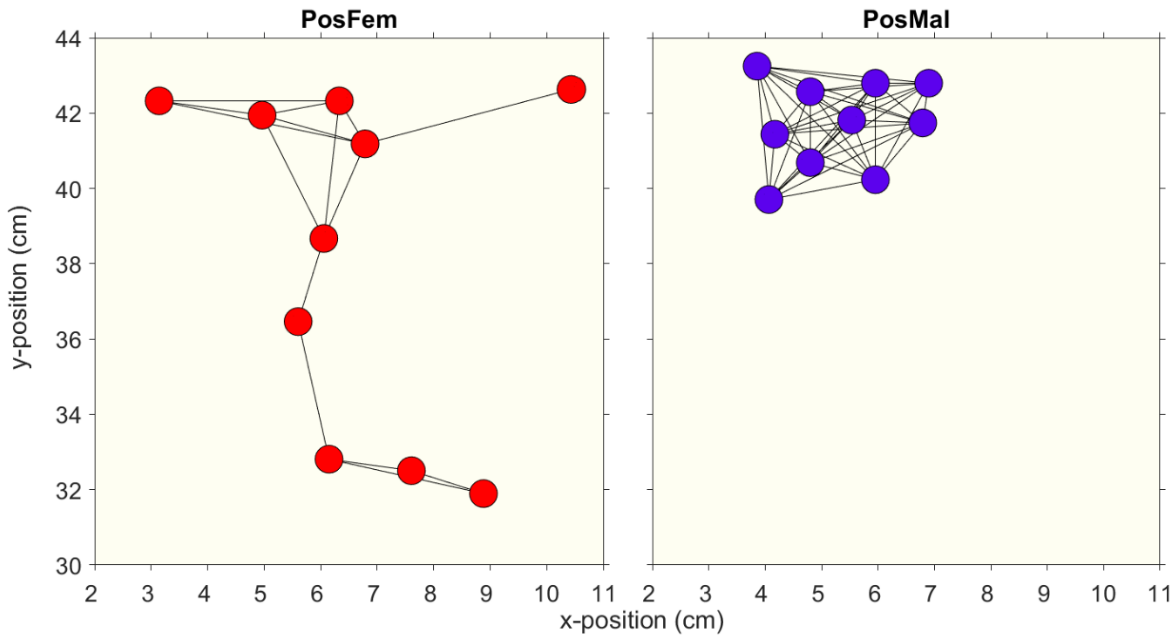

Figure S5. Vertical distribution of insects on the wall for the four conditions. Fraction of bugs by horizontal strips of 5 mm, from 0 to 440 mm, corresponding to the height of the setup for potentially weakly infected (females: pink, males: cyan) and infected bugs (females: red, males: blue)

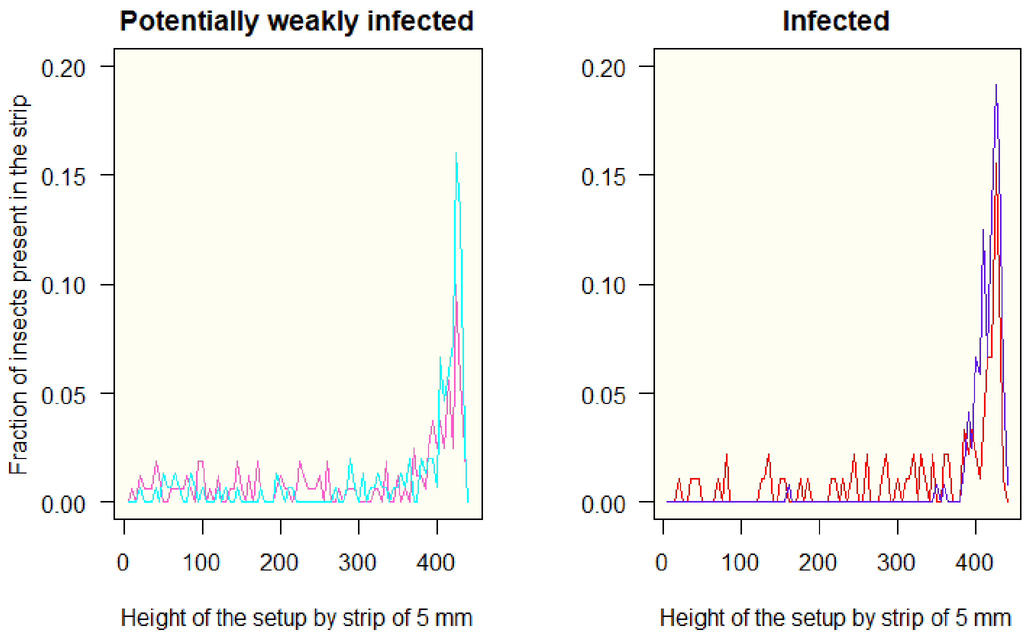

Supplementary material S6. Correlation between experimental conditions and the results from aggregation and geotaxis

S6.A. Values of the main index of aggregation (number of aggregated insects) and of geotaxis (mean vertical position Y of triatomines) for each experiment at 150 min, together with the conditions of temperature, relative humidity, and the time of the experiment (expressed in minutes calculated from the beginning of the photophase). The photophase started every day at 9 am when the light of the insectarium was switched on. During the experiments, the only light in used in the laboratory was the light of the setup. Data are given for the four experimental groups: potentially weakly infected females (PWFem) and males (PWMal), and infected females (PosFem) and males (PosMal).

Group	# aggregated insects	Mean Y position of triatomines	Temperature (°C)	Humidity (% RH)	Time (in min) from the beginning of the photophase
PWFem	7	405.34	25.6	64	42
PWFem	7	387.28	25.6	62	50
PWFem	3	158.04	25.5	72	55
PWFem	4	210.74	26.2	64	5
PWFem	6	294.48	26.4	49	4
PWFem	8	316.82	25.9	54	40
PWFem	4	179.59	26.2	57	45
PWFem	6	300.79	26.6	54	11
PWFem	5	240.69	25.5	52	30
PWFem	9	332.56	25.3	51	45
PWFem	2	269.01	24.9	49	45
PWFem	9	349.93	25.6	51	8
PWFem	8	370.55	25.2	52	28
PWFem	8	299.32	27.2	52	15
PWFem	9	279.68	26.6	61	27
PWFem	6	254.67	25.9	53	18
PWMal	8	382.32	25.9	62	15
PWMal	10	371.72	26.0	64	15
PWMal	10	89.57	27.6	62	5
PWMal	9	409.15	25.5	57	50
PWMal	4	404.74	25.7	53	10
PWMal	7	341.43	26.7	52	25
PWMal	7	413.36	27.2	51	20
PWMal	8	354.03	25.8	65	10
PWMal	10	406.11	25.5	60	35
PWMal	5	268.77	27.0	52	40
PWMal	9	377.31	26.5	49	20
PWMal	10	388.40	25.3	58	10
PWMal	7	246.92	25.4	52	5
PWMal	9	406.96	25.2	53	7
PWMal	9	421.89	26.9	47	40

PosFem	7	353.68	26.1	50	21
PosFem	9	413.31	27.4	52	13
PosFem	10	254.35	26.2	53	4
PosFem	5	371.02	27.0	51	5
PosFem	9	336.71	27.1	51	55
PosFem	9	306.22	27.3	54	13
PosFem	10	286.61	27.0	62	25
PosFem	8	382.75	24.9	61	15
PosFem	8	256.95	27.1	58	20
PosMal	10	413.03	26.5	51	30
PosMal	10	416.88	27.2	65	7
PosMal	10	423.01	27.2	51	10
PosMal	8	395.30	27.4	52	10
PosMal	10	417.02	27.5	54	22
PosMal	7	417.24	25.3	61	55
PosMal	9	419.26	27.0	55	6
PosMal	8	422.63	26.3	59	32
PosMal	9	415.56	27.4	61	30
PosMal	9	415.22	24.8	55	12
PosMal	9	373.46	27.6	55	45
PosMal	10	408.81	25.2	56	18

S6.B Linear regressions between the index of aggregation (number of aggregated triatomines) and index of geotaxis (mean vertical position Y of the triatomines) and each laboratory condition: temperature, relative humidity and time of the experiment (expressed in minutes after the beginning of the photophase) at 150 minutes, for each group (potentially weakly infected females (PWFem) and males (PWMal), and infected females (PosFem) and males (PosMal)) and for all the experiments together.

		Temperature (°C)	Relative humidity (RH)	Time (min) from photophase
PWFem	*mean±SD*	*25.9 ± 0.6*	*56.1 ± 6.6*	*29.3 ± 17.3*
	Linear regression Aggregation	P = 0.41	P = 0.35	P = 0.42
	Linear regression Y position	P = 0.52	P = 0.31	P = 0.92
PWMal	*mean±SD*	*26.1 ± 0.8*	*55.8 ± 5.7*	*20.5 ± 14.4*
	Linear regression Aggregation	P = 0.66	P = 0.14	P = 0.97
	Linear regression Y position	P = 0.08	P = 0.45	P = 0.24
PosFem	*mean±SD*	*26.7 ± 0.8*	*54.7 ± 4.5*	*19.0 ± 15.2*
	Linear regression Aggregation	P = 0.79	P = 0.36	P = 0.53
	Linear regression Y position	P = 0.70	P = 0.45	P = 0.98
PosMal	*mean±SD*	*26.6 ± 1.0*	*56.2 ± 4.4*	*23.1 ± 15.7*
	Linear regression Aggregation	P = 0.44	P = 0.42	P = 0.09
	Linear regression Y position	P = 0.37	P = 0.50	P = 0.36
All	*mean±SD*	*26.3 ± 0.8*	*55.8 ± 5.4*	*23.5 ± 15.8*
	Linear regression Aggregation	P = 0.08	P = 0.93	P = 0.14
	Linear regression Y position	P = 0.81	P = 0.24	P = 0.96

S6.C. Boxplot of the temperature, relative humidity, and time from the beginning of the photophase (min) for the four groups: potentially weakly infected females (PWFem) and males (PWMal), and infected females (PosFem) and males (PosMal). Results of Anderson-Darling all-pairs comparison tests are shown at the top of the figure (same letters correspond to groups no-statistically significantly different at a *p*-value of 0.05).

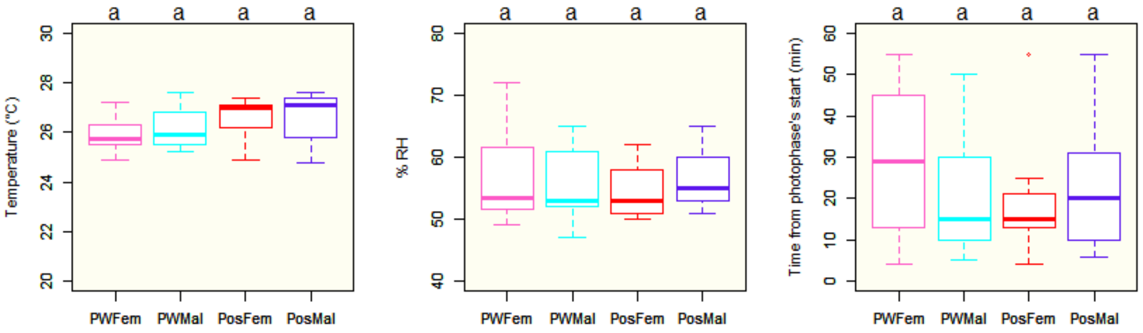

Supplement: Supplementary file 1 — Supplementary material [file 41598_2019_53966_MOESM1_ESM.pdf]
